# Bibliometric analysis and visualization of the research on the relationship between RNA methylation and immune cell infiltration in tumors

**DOI:** 10.3389/fimmu.2024.1477828

**Published:** 2024-12-12

**Authors:** Sibo Meng, Guanghui Yang, Enhao Yu, Jiaxin Li

**Affiliations:** ^1^ Qilu Hospital, Cheeloo College of Medicine, Shandong University, Qingdao, Shandong, China; ^2^ Qilu Hospital of Shandong University, Cheeloo College of Medicine, Shandong University, Jinan, Shandong, China

**Keywords:** bibliometric analysis, RNA methylation, immune cell, web of science, trend topics

## Abstract

**Background:**

This research endeavors to delve into the research hotspots and trends concerning RNA methylation and tumor immune cells through the application of bibliometric analysis and visualization techniques.

**Methods:**

A comprehensive search in WoSCC (2014-2023) for RNA methylation and tumor immune cell articles/reviews was conducted. Bibliometric analysis and visualization employed CiteSpace, Bibliometric, and VOSviewer tools.

**Results:**

A total of 3295 articles were included in the analysis, with a continuously increasing number of publications linking RNA methylation to tumoral immune cells. Chinese authors and research institutions have demonstrated a sustained growth trend in both the number of publications and author influence. SUN YAT SEN UNIVERSITY, a Chinese institution, has published the highest number of articles in this field, while also demonstrating extensive international and inter-institutional collaborations. Meanwhile, HARVARD UNIVERSITY has also achieved impressive results. For instance, Frontiers in Immunology has published the largest number of articles in this category. Nature Communications has published articles that are most influential in this field, playing a pivotal role in disseminating research findings. The sustained vitality of this field is attributed to its solid research foundation, including the groundbreaking work published by Professor Chiappinelli KB in Cell and the widely cited paper by Professor Han DL in Nature. Analysis of research trend topics reveals that m5C, immunotherapy, and the immune microenvironment are current research focuses.

**Conclusion:**

Future investigative efforts at the juncture of RNA methylation and tumor immune cells are anticipated to concentrate on domains including m5C, n7-methylguanosine, cuproptosis, prognosis assessment, immunotherapeutic strategies, and the tumor microenvironment.

## 1 Introduction

Currently, over 170 types of RNA modifications are known (1), this includes RNA methylation and other chemical modifications represented by acetylation such as AC4C. Among them, RNA methylation studies constitute the majority, encompassing modifications such as m1A, m6A, m5C, m7G, m3C, etc. (2), which modify the AUGC bases and function as post-transcriptional regulators. They exhibit dynamic equilibrium characteristics and involve corresponding ‘writers’, ‘erasers’, and “readers” (3). These modified RNAs participate in various biological processes, including but not limited to RNA splicing, translation, transport, and RNA stability, influencing RNA production, transport, function, and metabolic processes. Based on these molecular functions, RNA modifications are involved in multiple biological processes such as cell development, differentiation, activation, migration, and polarization. Over the past decade, research has shown that RNA modifications play a crucial role in the occurrence, development, and drug resistance of various solid tumors and hematological malignancies (4–8). Numerous literature sources have confirmed that RNA modifications participate in tumorigenesis and the tumor-associated immune environment (9). However, the interaction network between the complexity of RNA modifications and the diversity of immune cells still needs to be further elucidated (10). Targeting RNA modifications for the treatment of immune-related diseases remains at the theoretical stage, with no clinical application examples currently available. Therefore, our research topic holds significant value.

The Tumor Microenvironment (TME) plays a role in tumor initiation, progression, invasion, and metastasis, yet our understanding of its specific mechanisms remains inadequate. Immune cells are a vital component of the TME. RNA methylation exerts a pivotal function in both innate and adaptive immune responses, encompassing macrophage polarization, promoting the accumulation of Myeloid-Derived Suppressor Cells (MDSCs), influencing the function of dendritic cells in antigen presentation, reducing the infiltration and activation of effector T cells, modulating the differentiation of regulatory T cells (Tregs), and promoting abnormal proliferation of B cells. Macrophages are involved in tumorigenesis, metastasis, and drug resistance (11). MDSCs suppress T-cell-mediated immune responses, impacting cancer prognosis (12). The function and activity of dendritic cells are influenced by immunosuppressive factors, potentially leading to immune evasion (12). RNA methylation regulates T-cell proliferation, activation, apoptosis, and other functions through modulating factors (13). RNA m6A methylation plays various roles in B-cell development, maturation, and antibody secretion (13). Additionally, immune-therapeutics also rely on the function of immune cells, and inhibiting RNA methylation in combination with immunotherapy drugs has preliminarily shown increased efficacy (14).

In the investigation of the causes of tumorigenesis, RNA methylation and immune cells are both hot research topics, and numerous articles have reported their intricate connections. However, to our knowledge, bibliometric analysis on this subject remains lacking. Bibliometrics, through quantitative analysis of data and intuitive visualization, reveals research trends and hot topics. It assesses the authority of published literature by examining citation counts and international/inter-institutional collaborations, providing information for research decision-making. Therefore, we have conducted a bibliometric analysis of the literature related to immune cells and RNA methylation over the past decade, comprehensively showcasing the global research status in this field and summarizing current hot topics.

## 2 Materials and methods

### 2.1 Data source and collection

**Figure 1:**
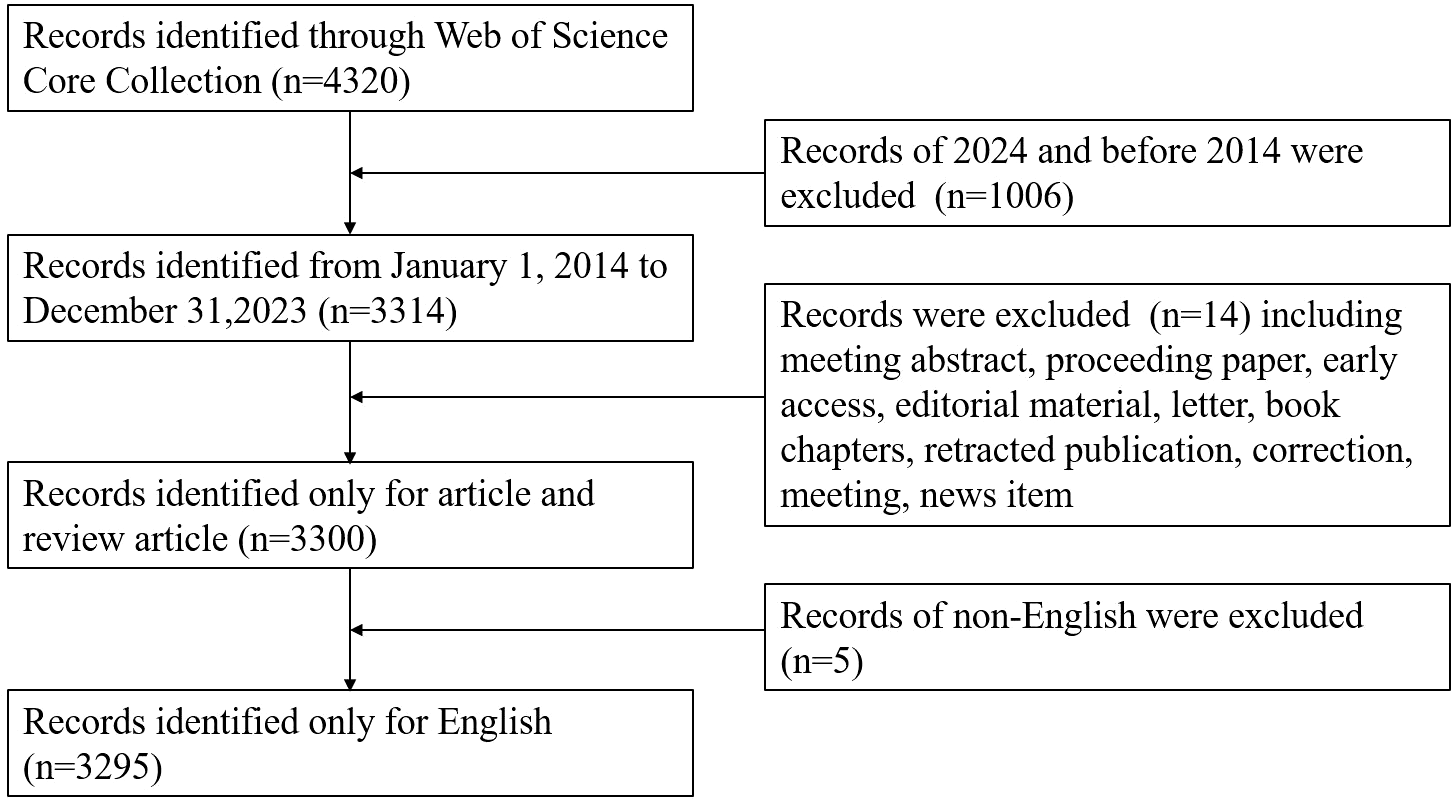
Flowchart for the screening process.

In order to mitigate bias, we conducted a literature search in the Web of Science on July 14, 2024, using the Topic (TS) to identify articles related to RNA methylation AND Immune cell AND Tumor. The search terms used are outlined in **Table 1**. The search results excluded articles published in 2024, were restricted to English language only, and were limited to articles and reviews. J.L. and S.M. conducted the search independently, and a total of 3295 articles from 2014 to 2023 were exported in the “full record with cited references” format from Plain Text File for final analysis (**Figure 1**).

**Table 1:** Logical relationship between search terms.

Number	Topic	Terms
#1	RNA methylation	(((((((((((((((((((((((((((((TS=(N6-methyladenosine)) OR TS=(m6A methylation)) OR TS=(“m (6)A”)) OR TS=(m6A modification)) OR TS=(N-6-methyladenosine)) OR TS=(“adenosine N6 methylation”)) OR TS=(6-methyladenine)) OR TS=(5-methylcytosine)) OR TS=(m5C methylation)) OR TS=(N1-methyladenosine)) OR TS=(m1A methylation)) OR TS=(N3-methylcytosine)) OR TS=(m3C methylation)) OR TS=(N7-methylguanosine)) OR TS=(m7G methylation)) OR TS=(5-methyluridine)) OR TS=(m5U methylation)) OR TS=(N2-methylguanosine)) OR TS=(m2G methylation)) OR TS=(2’-O-methyladenosine)) OR TS=(2’-O-methylation)) OR TS=(2’-O-Me)) OR TS=(2’-O-RNA)) OR TS=(m6am methylation)) OR TS=(5-hydroxymethylcytosine)) OR TS=(hmsc methylation)) OR TS=(pseudouridine)) OR TS=(pseudouridylation)) OR TS=(Ψ)) OR TS=(RNA methylation)
#2	Immune cell	(((((((((((TS=(immune AND cell*)) OR TS=(b AND cells*)) OR TS=(t AND cells*)) OR TS=(plasma AND cell*)) OR TS=(macrophage*)) OR TS=(nk AND cell*)) OR TS=(monocyte*)) OR TS=(dendritic AND cell*)) OR TS=(mast AND cell*)) OR TS=(neutrophil*)) OR TS=(natural AND killer AND cell*)) OR TS=(lymphocyte*)
#3	Tumor	(((((((TS=(cancer)) OR TS=(tumor)) OR TS=(carcinoma)) OR TS=(neoplasm)) OR TS=(tumorous)) OR TS=(neoplastic)) OR TS=(malignancy)) OR TS=(malignant tumor)
#4	Total	#1 AND #2 AND #3

### 2.2 Bibliometric analysis and visualization

We conducted statistical analysis on the included literature, utilizing Excel and Word within Office 2019 to analyze trends in annual publication volumes, most publications countries, institutions, authors, and journals that have published the most on this topic, as well as highly cited documents. We leveraged software such as VOSviewer (version 1.6.20) (15), R package Bibliometrix (version 4.3.0) (www.bibliometrix.org) (16), and CiteSpace (version 6.3.R1) (17) to conduct collaboration, co-occurrence, citation, and coupling analyses on the aforementioned countries, institutions, authors, and journals, and presented the findings through charts and diagrams. We also performed keywords clustering and keywords burst analysis, and used graphics to illustrate recent research hotspots and trends through timelines, Thematic-evolution-Map, and trending topics. Through these comprehensive analyses, we presented a thorough overview of the evolution and current status of this research field.

## 3 Results

### 3.1 An overview of publication trends and main information

**Figure 2:**
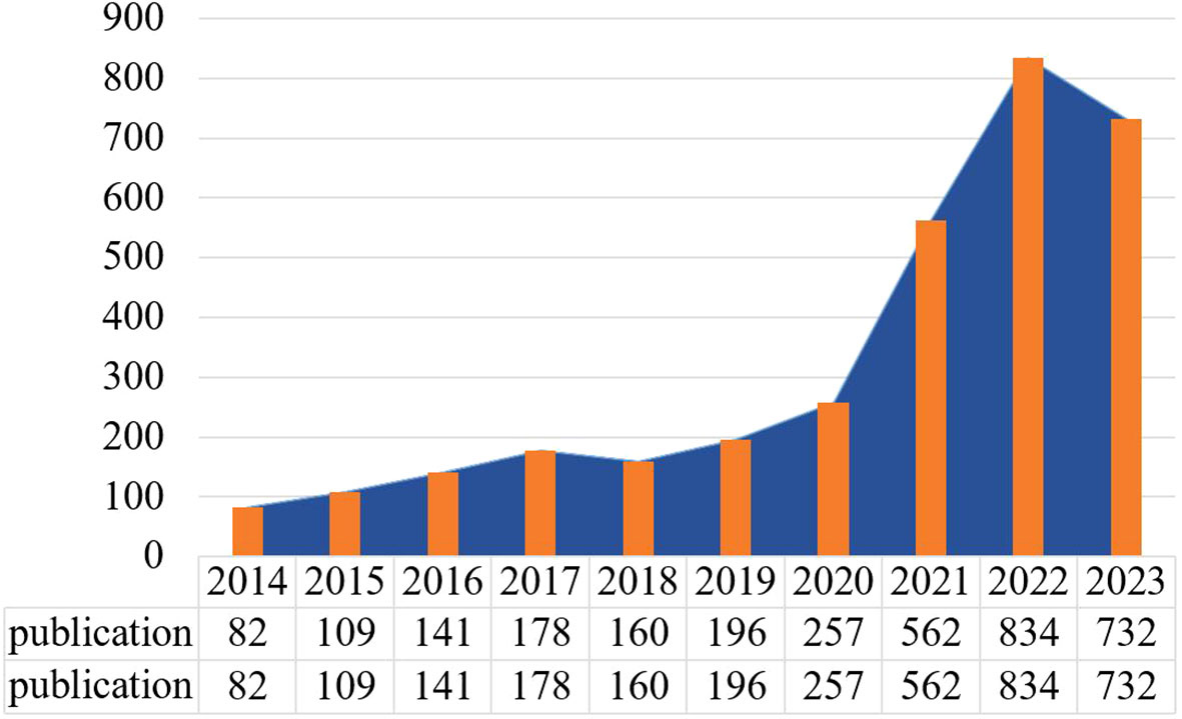
An overview of the annual number of publications.

Over the past 10 years, publications in the field of tumor research on RNA methylation and immune cells have increased annually, demonstrating a pronounced upward trend (**Figure 2**). The annual publication volume has risen from 82 in 2014 to 834 in 2022, signifying a continually growing interest in this area. Notably, after 2021, the growth rate of literature in this field accelerated. While there was a slight decrease in 2023 (n=723), it remained at a relatively high level. Among the included literature, there are 819 sources, involving a total of 21,962 authors, 2,713 articles accounting for 82.3%, 582 reviews accounting for 17.7%, 5,314 author’s keywords, and 137,372 references (**Supplementary Table 1**).

### 3.2 Authors and co-authorship analysis

**Figure 3:**
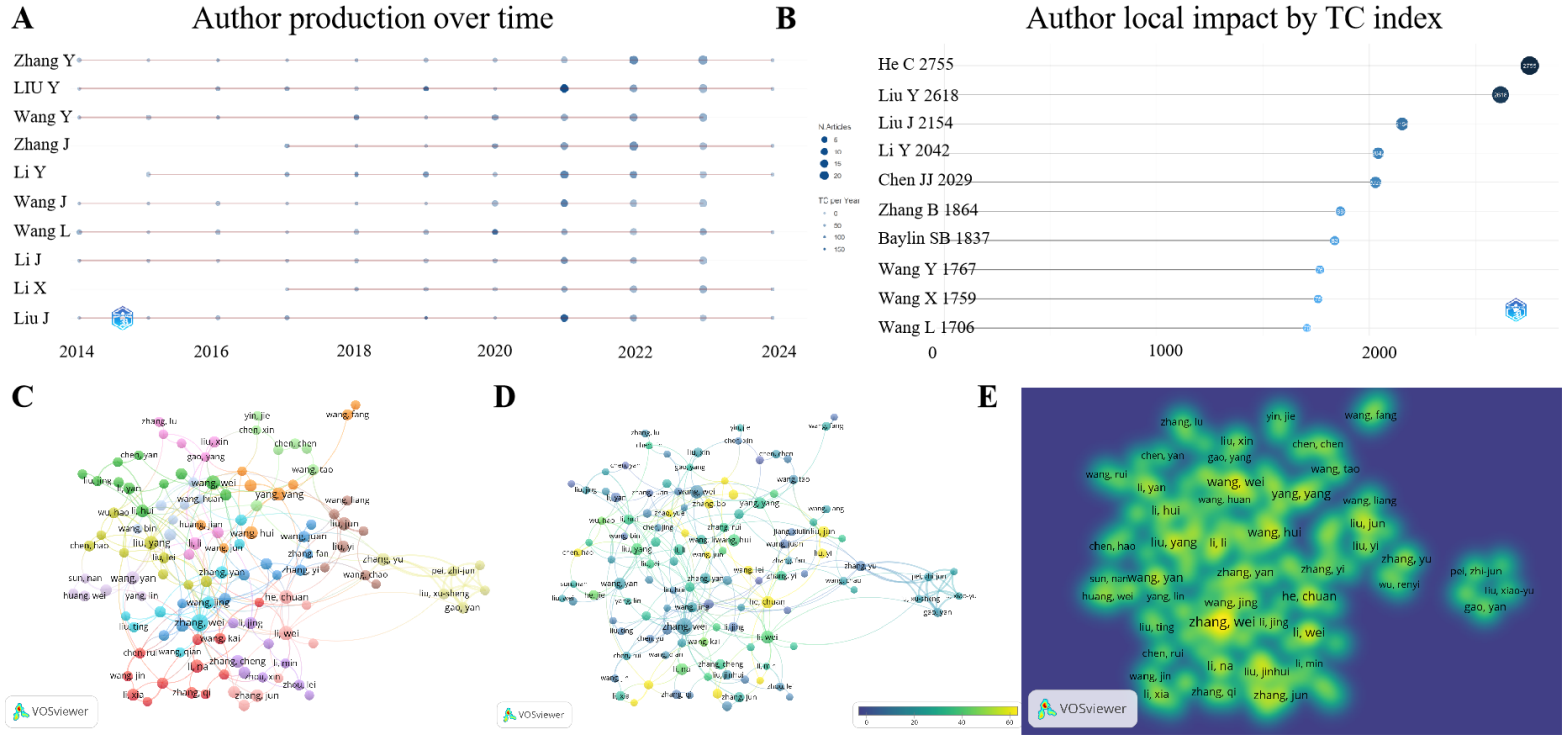
Authors and co-authorship analysis. **(A)** is the annual statistical publication of the top 10 authors, **(B)** is the total author impact ranking, and **(C–E)** are respectively the network, overlay and density documents views about co-authorship in the VOSviewer website, the closer to yellow indicates a higher number of publications.

We first examined the number of publications by authors. It is evident that the top 10 authors with the highest number of publications in the past decade are all from China, with Professor ZHANG Y leading the pack with 63 publications. The total number of publications by these top 10 authors over the decade exceeds 40 (**Supplementary Table 2**). Furthermore, an analysis of their annual publication rates indicates a notable increase in publications over the past three years, mirroring the overall trend (**Figure 3A**). In terms of author influence, based on metrics such as h-index, g-index, m-index and publication count, the top 10 authors do not entirely overlap (**Supplementary Figures 1A-D**). Summarizing these three indicators, Professor HE C emerges as the most author impact, ranking 27th in total publications but having published earlier in the field and garnered a high number of citations, reflecting significant impact and recognition (**Figure 3B**, **Table 2**, **Supplementary Table 3**).

**Table 2:** Top 10 author impact (TC ranking).

Author	H-index	G-index	M-index	TC	NP	PY_start
He C	16	26	1.778	2755	26	2016
Liu Y	21	51	1.909	2618	60	2014
Liu J	16	39	1.455	2154	39	2014
Li Y	19	45	1.9	2042	49	2015
Chen JJ	14	18	1.75	2029	18	2017
Zhang B	11	18	1.375	1864	18	2017
Baylin SB	6	6	0.6	1837	6	2015
Wang Y	19	41	1.727	1767	56	2014
Wang X	16	39	1.455	1759	39	2014
Wang L	17	41	1.545	1706	45	2014

There is also a strong interconnection among authors through co-authorships. In terms of the number of collaborative publications, Professor Zhang W tops the list with 30 articles (**Figures 3C-E**, **Table 3**, **Supplementary Table 4**). However, a comprehensive assessment that takes into account both the quantity of publications and their average citation rate yields a different ranking (**Supplementary Figures 2A-C**).

**Table 3:** Top 10 co-authorship (Documents ranking).

Author	Documents	Citations	Total link strength
Zhang W	30	77	11
Wang W	19	426	7
Li W	19	200	6
He C	17	364	8
Liu Y	16	281	6
Yang Y	15	201	7
Wang Y	15	109	3
Wang H	15	7	1
Li N	15	474	0
Liu J	14	20	5

### 3.3 Productive institutions analysis

**Figure 4:**
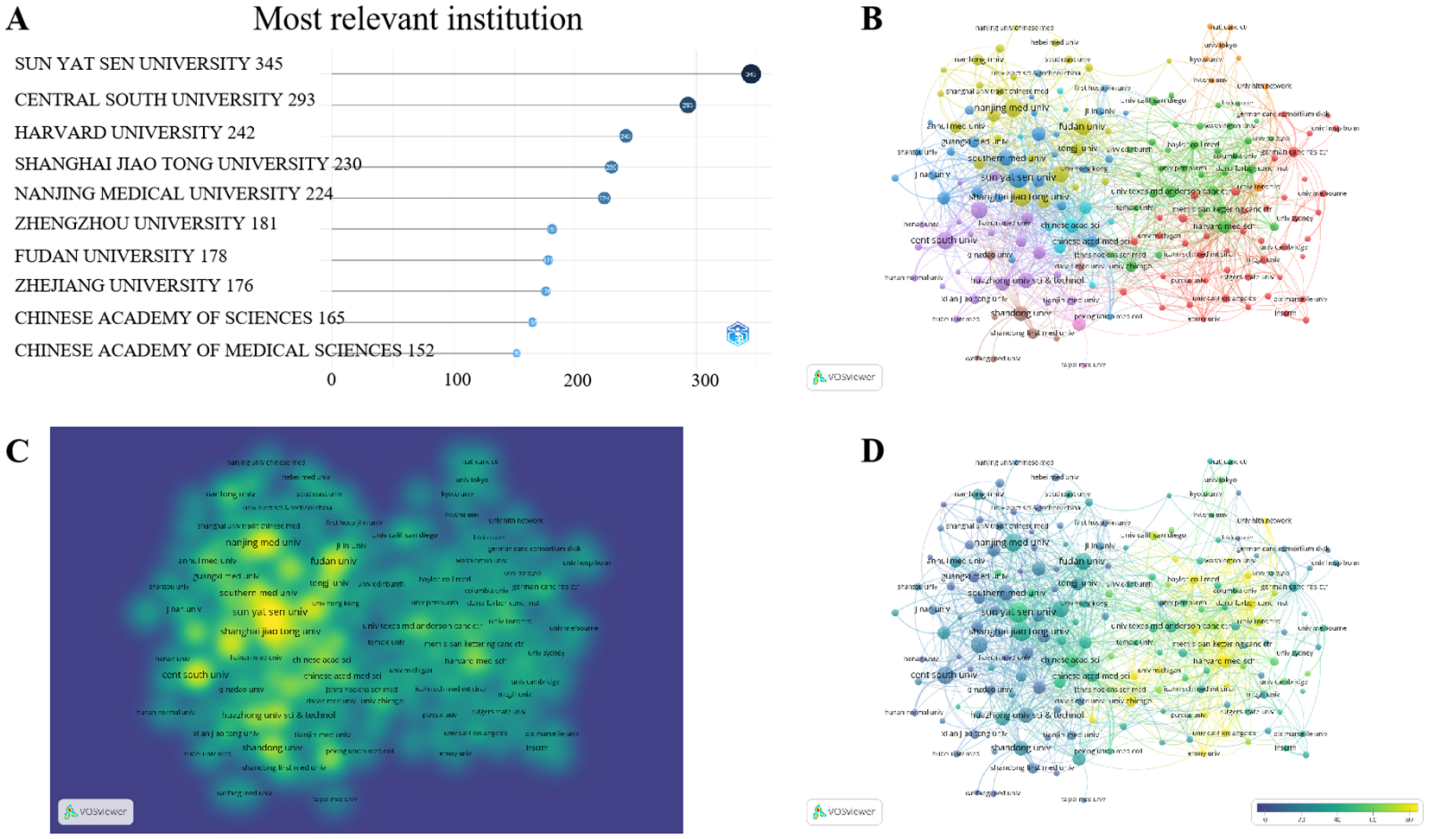
Institutions publications analysis. **(A)** represents the total number of publications from the top ten institutions, **(B, C)** represent the collaboration among institutions, and **(D)** represents the average number of collaborative publications per institution in the VOSviewer website, the closer to yellow indicates a higher number of publications.

Our analysis of institutional publication data reveals that nine of the top ten institutions publishing articles in this field over the past decade are from China, with one being from the United States. SUN YAT SEN UNIVERSITY leads the pack, having accumulatively published 345 documents, while the top ten institutions collectively published over 150 articles during this period (**Figure 4A**, **Supplementary Table 5**). Examining the annual publication trends, HARVARD UNIVERSITY held the top position prior to 2021, but has since been surpassed by SUN YAT SEN UNIVERSITY and CENTRAL SOUTH UNIVERSITY in 2022, signifying the latter two institutions’ intensified research efforts in this field over the past three years (**Supplementary Figure 3A**).

Collaborations among institutions underscore their close relationships and interconnectedness. SUN YAT SEN UNIVERSITY tops the list, having collaborated with 146 institutions to produce 141 articles (**Figures 4B, C**, **Supplementary Figure 3A**, **Supplementary Table 6**). However, it’s essential to note that while HARVARD UNIVERSITY may not have the highest number of collaborative publications, it boasts the highest average citation count per article, indicative of the exceptional quality of its research outputs (**Figure 4D**, **Supplementary Figure 3B**).

### 3.4 Analysis of countries publications

**Figure 5:**
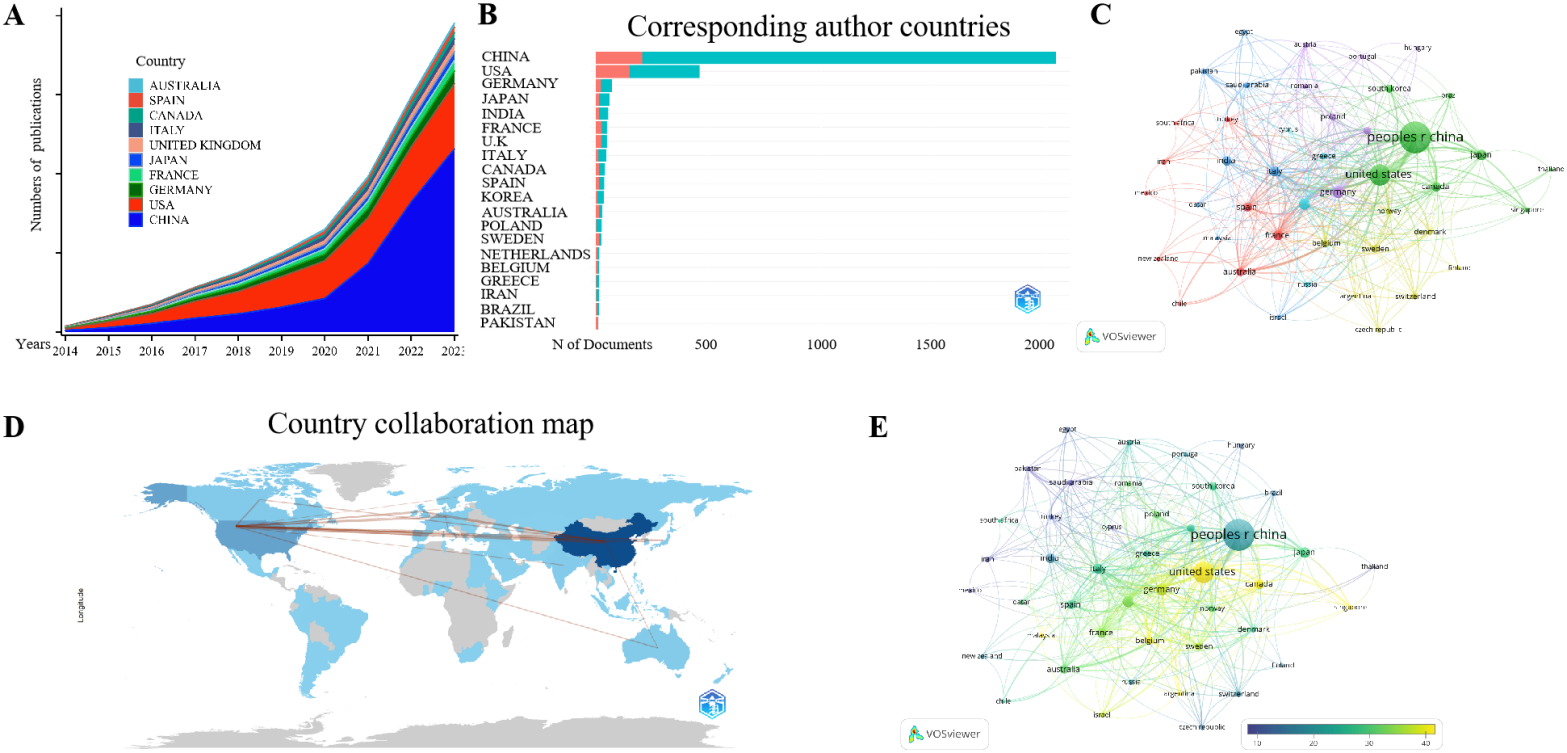
Analysis of the number of national publications. **(A)** is a stacked chart of the top ten countries with the largest number of publications in the past ten years. **(B)** shows the number of publications by the country of the corresponding author. Blue represents publications solely completed by domestic authors, while red represents publications completed through international collaborations. **(C, D)** illustrate the international cooperation among countries. The depth of color in **(D)** is positively correlated with the number of publications by the country. **(E)** represents the average number of citations per publication by country, with yellow indicating a higher average number of citations.

Furthermore, we conducted an analysis on the number of publications by countries. A total of 75 countries/regions authors have published articles in this field, with CHINA and the USA far ahead in terms of the number of authors publishing in this field, with a cumulative total of 7,074 and 2,498 publications respectively over a period of ten years (**Figure 5A**, **Supplementary Table 7**). When counting by the nationality of the corresponding author, CHINA ranks first with 2,043 articles (**Supplementary Figure 4A**). Similar to the number of publications by institutions, the number of publications by the USA has been slowly increasing year by year, making it the country with the most publications in this field worldwide before 2020. However, CHINA has rapidly risen in this field since 2021, becoming the country with the largest number of publications (**Supplementary Figure 4B**). Among these, 62% are Articles, with 207 being completed through collaborations between Chinese and foreign authors (MCP), accounting for approximately 10.1%, and 1,836 articles being solely completed by Chinese authors (SCP) (**Figure 5B**, **Supplementary Figure 4C**, **Table 4**, **Supplementary Table 8**). From the perspective of international collaboration, CHINA, the USA, and several European countries have more collaborations (**Figures 5C, D**). Additionally, it is worth mentioning that the annual number of publications by CHINESE authors also leads the world (**Supplementary Figure 4D**), with the number of citations far exceeding that of other countries, reaching 42,203 times. However, when calculating the average number of citations per article, USA authors have a higher average than Chinese authors (**Figure 5E**, **Supplementary Table 9**).

**Table 4:** Top 10 countries publications (Corresponding author ranking).

Country	Articles	Articles %	SCP	MCP	MCP %
CHINA	2043	62	1836	207	10.1
USA	462	14	310	152	32.9
GERMANY	72	2.2	48	24	33.3
JAPAN	61	1.9	46	15	24.6
INDIA	56	1.7	42	14	25
FRANCE	51	1.5	25	26	51
UNITED KINGDOM	51	1.5	26	25	49
ITALY	47	1.4	35	12	25.5
CANADA	42	1.3	27	15	35.7
SPAIN	38	1.2	22	16	42.1

### 3.5 Analysis of journals

**Figure 6:**
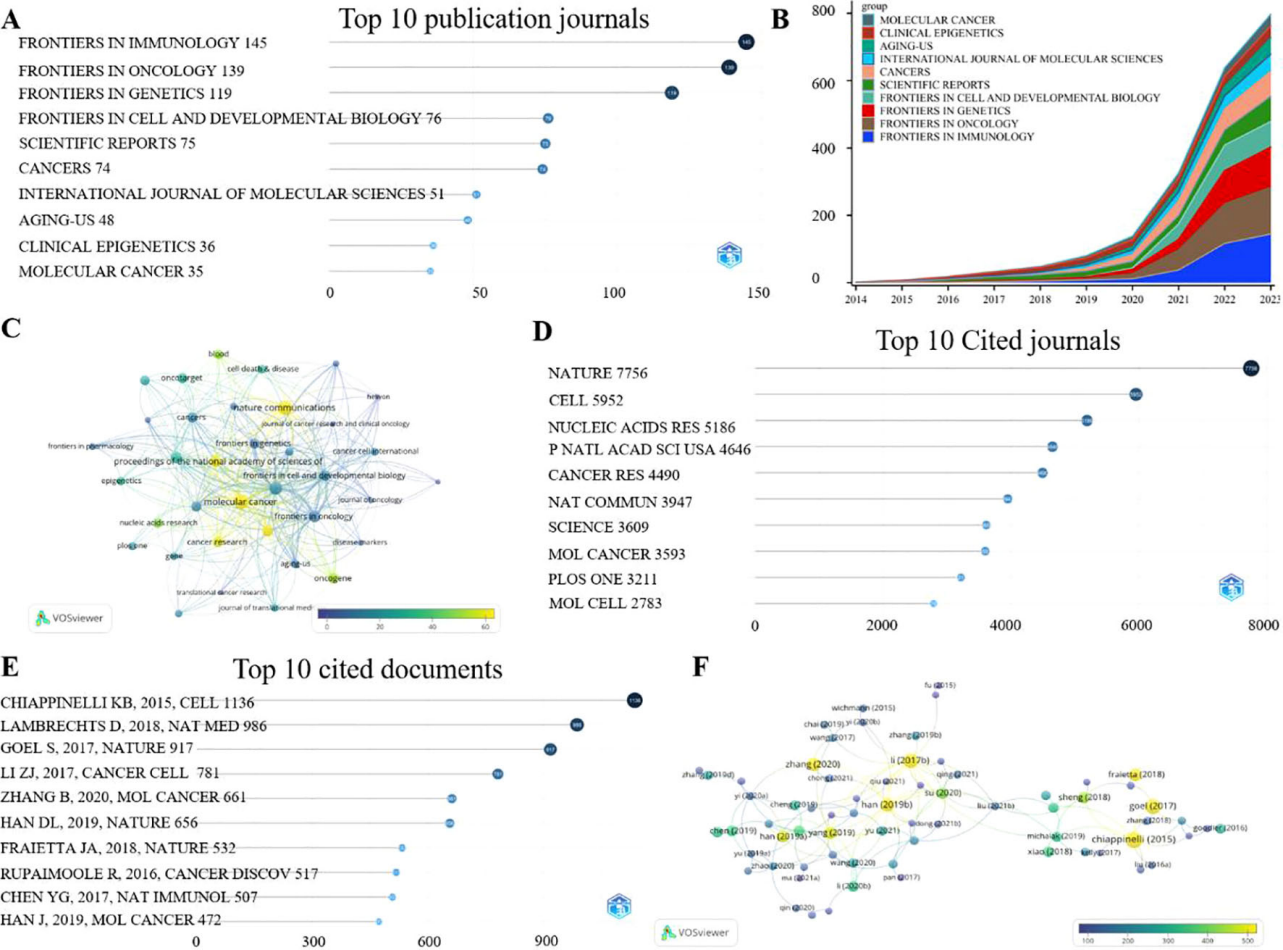
Analysis of publish journals and highly cited literatures. **(A)** displays the top 10 journals with the highest number of publications, **(B)** shows the annual change in publication volume for the top 10 journals, **(C)** adjusts the publication volume of journals based on the number of citations, with closer to yellow indicating more citations, **(D)** displays the top 10 journals that are most frequently cited as references, **(E)** shows the top 10 cited article titles and citation numbers, **(F)** shows the authors of the highly cited articles. Closer to yellow indicates a higher average number of citations per article.

The analysis of journals publishing articles is divided into two parts. The first part focuses on analyzing the journals that publish the most in this field, while the second part investigates which journals are cited as supporting evidence in the articles within this field. Among the 3,295 published articles included in our analysis, they were disseminated across 819 journals (**Supplementary Table 10**). FRONTIERS IN IMMUNOLOGY stands out as the journal with the highest number of publications in this field, having published a total of 145 articles related to the field over a span of 10 years (**Figure 6A**). The top 10 journals by publication volume have published at least 35 articles each, with a continuous increase in their publication rates (**Figure 6B**, **Supplementary Table 10**). Beyond mere publication counts, we also considered the journal impact through metrics such as the H-index, G-index, and M-index (**Figures 5A-C**, attached). Based on the Bradford Laws, we identified the top 10 journals with the highest core impact in this field (**Supplementary Figure 5D**). Although NATURE COMMUNICATIONS does not rank among the top 10 in terms of total publication volume, it exhibits significant journal impact (**Table 5**, **Supplementary Table 11**). Additionally, journals like Mol Cancer, P Natl Acad Sci Usa, and Cancer Res also boast high average citation counts per article (**Figure 6C**, **Supplementary Table 12**). Upon analyzing the cited journals, we found that articles from 44 journals have been cited over 1,000 times, with NATURE leading the pack with 7,756 citations (**Figure 6D**, **Supplementary Table 13**).

**Table 5:** Top 10 journals impact (Total ranking).

Source	H-index	G-index	M-index	Total	Articles	IF	JCR
Nat Commun	25	31	2.273	3349	31	14.7	Q1
Mol Cancer	23	35	2.3	3630	35	27.7	Q1
Front Immunol	22	38	2.2	2203	145	5.7	Q1
Front Oncol	20	30	2.222	1501	139	3.5	Q2
Oncotarget	20	30	1.818	945	34	—	—
P Natl Acad Sci Usa	20	24	2	1647	24	9.4	Q1
Cancers	19	27	2.714	1011	74	4.5	Q1
Oncogene	18	22	1.8	1102	22	6.9	Q1
Cancer Res	17	21	1.7	1182	21	12.5	Q1
Clin Epigenetics	17	27	1.7	795	36	4.8	Q1

### 3.6 Classic article analysis

The number of citations indicates the influence of a particular literature within its field. Analyzing the number of journals cited globally in this field, Professor CHIAPPINELLI KB’s article published in CELL in 2015 has the highest citation count of 1136, with an average of 113 citations per year (**Figure 6E**, **Table 6**, **Supplementary Table 14**). If we limit the analysis to the 3295 articles included in our study, Professor HAN DL’s article published in NATURE in 2019 has been cited 238 times (**Supplementary Figure 6A**, **Supplementary Table 15**). Among the highly cited articles, Chinese authors have a higher number of citations (**Figure 6F**), which may be proportional to their publication volume (**Supplementary Figure 6B**). However, in the references cited within these highly cited articles, foreign institutions and authors account for a considerable proportion (**Supplementary Figures 6C, D**).

**Table 6:** Top 10 citations publication (Total Citations TC ranking).

Cited papers	Global Citation	TC per Year	Local Citation	LC/GC Ratio
CHIAPPINELLI KB, 2015, CELL (18)	1136	113.60	91	8.01
LAMBRECHTS D, 2018, NAT MED (19)	986	140.86	11	1.12
GOEL S, 2017, NATURE (20)	917	114.63	16	1.74
LI ZJ, 2017, CANCER CELL (21)	781	97.63	92	11.78
ZHANG B, 2020, MOL CANCER (22)	661	132.20	221	33.43
HAN DL, 2019, NATURE (23)	656	109.33	238	36.28
FRAIETTA JA, 2018, NATURE (24)	532	76.00	15	2.82
RUPAIMOOLE R, 2016, CANCER DISCOV (25)	517	57.44	4	0.77
CHEN YG, 2017, NAT IMMUNOL (26)	507	63.38	12	2.37
HAN J, 2019, MOL CANCER (27)	472	78.67	73	15.47

### 3.7 Analysis of co-citation and coupling

The strength of the relationship between documents can be reflected by Co-citation and Coupling. The article published by Professor Jemal A in the CA Cancer J Clin journal in 2011 (28) had the highest Co-citation (**Supplementary Table 16**). The article published by Professor Chiappinelli KB in CELL in 2015 (18) had the highest Coupling (**Supplementary Figure 7A**, **Supplementary Table 17**). The journal with the highest Co-citation is NATURE (**Supplementary Figure 7B**, **Supplementary Table 18**), the journal with the highest Coupling documents is Frontiers in Immunology (**Supplementary Figure 7C**), and the journal with high Coupling citations is Molecular Cancer (**Supplementary Figure 7D**, **Supplementary Table 19**). This provides a method for identifying core documents, research hotspots, and frontier issues within disciplines, as well as assessing the influence of journals.

### 3.8 Analysis of keywords

**Figure 7:**
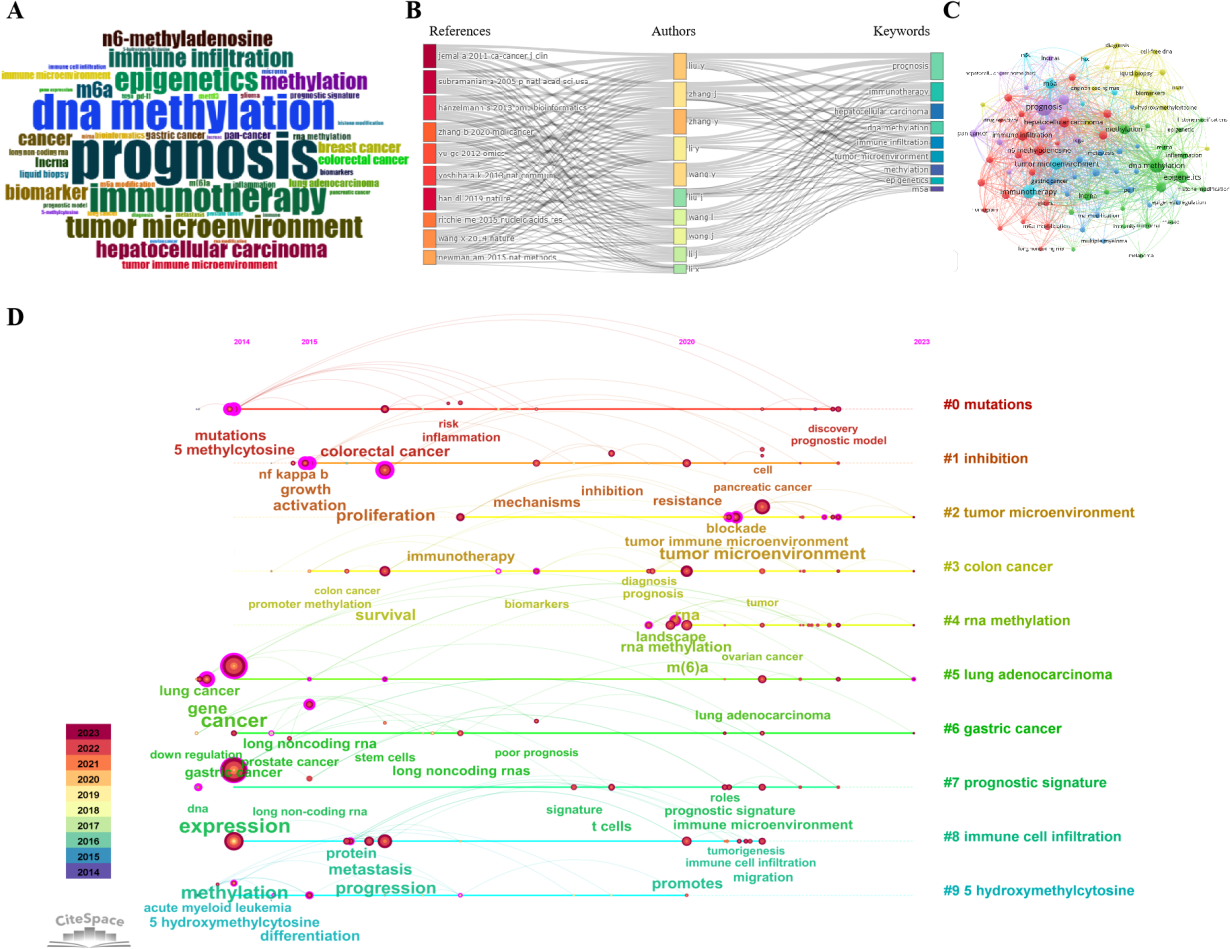
Analysis of keywords. **(A)** represents popular keywords, and the larger the symbol, the higher the frequency. **(B)** is a three-field diagram, reflecting the connection between highly cited articles, authors, and keywords. **(C)** represents the connection between popular keywords. **(D)** represents keyword clustering, and the timeline shows the popularity of keywords.

Keywords serve as the most fundamental concepts in an article, encapsulating its research direction or theme. Thus, a focused study on keywords can reveal significant shifts in research trends within a particular field. The articles included in our analysis encompass a total of 5,314 keywords (**Supplementary Table 20**), with “prognosis” being used 401 times by authors, indicating its popularity as a research topic in the realm of RNA methylation and immune cells. By examining the emergence timeline of these keywords and conducting a Keywords Bursts analysis, we discovered that “RNA methylation” and “m6A modification” emerged as the hottest keywords after 2021 (**Figure 7**, **Table 7**). Further analysis using a Treemap visualization based on sub-category proportions revealed that “expression” accounted for the largest share (**Supplementary Figure 8A**). Consistent with previous findings, among the most frequently cited articles, “prognosis” and the currently popular immunotherapy approach, “immunotherapy,” were also the most prevalent keywords (**Figure 7B**). These keywords were also commonly observed among Chinese authors with the highest publication output and in journals that frequently publish in this field (**Supplementary Figures 8B, C**). A Co-occurrence Network analysis of keywords unveiled a high frequency of co-occurrence between “prognosis,” immunotherapy, tumor microenvironment, immune cell infiltration, and methylation, underscoring the close relationships among these concepts (**Figure 7C**, **Supplementary Figures 8D-F**). Clustering the research on these keywords allows them to be divided into 10 categories, with Q=0.8385 and S=0.937, indicating good discrimination and strong internal consistency. Timeline analysis reveals that “#0 mutation” contains the most keywords, followed by “#1 inhibition” and “#2 tumor microenvironment” (**Figure 7D**). This suggests potential molecular mechanisms through which RNA modifications may impact tumor immune cells and the microenvironment. This underscores the fact that our chosen topic-RNA methylation and immune cells-represents one of the current hotspots in research.

**Table 7:** Top 20 Keywords with the Strongest Citation Bursts.

Keywords	Year	Strength	Begin	End	2014 - 2023
tumor suppressor	2014	18	**2014**	2018	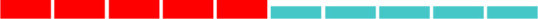
down regulation	2014	17.72	**2014**	2018	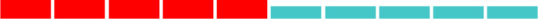
5 hydroxymethylcytosine	2014	16.88	**2014**	2020	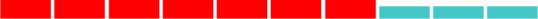
5 methylcytosine	2014	14.28	**2014**	2020	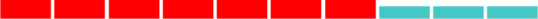
nf kappa b	2014	13.95	**2014**	2020	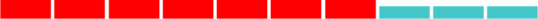
prostate cancer	2014	13.04	**2014**	2020	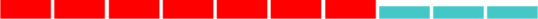
promoter methylation	2014	11.76	**2014**	2020	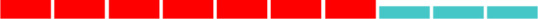
hypermethylation	2014	11.76	**2014**	2020	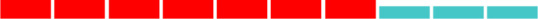
hypomethylation	2014	10.92	**2014**	2017	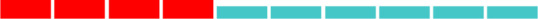
acute myeloid leukemia	2014	10.86	**2014**	2019	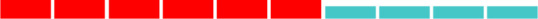
*in vivo*	2014	10.7	**2014**	2018	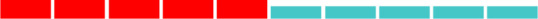
embryonic stem cells	2014	9.36	**2014**	2017	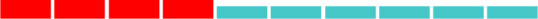
apoptosis	2015	11.26	**2015**	2021	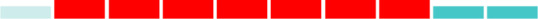
stem cells	2016	15.49	**2016**	2020	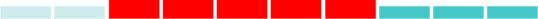
dna methylation	2014	16.79	**2017**	2019	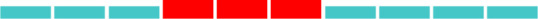
reveals	2017	11.85	**2017**	2021	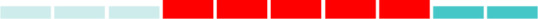
tumor microenvironment	2021	17.39	**2021**	2023	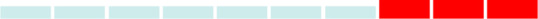
n6 methyladenosine	2021	11.78	**2021**	2023	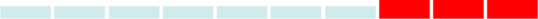
m(6)a modification	2021	10.65	**2021**	2023	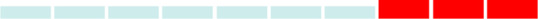
rna methylation	2020	9.56	**2021**	2023	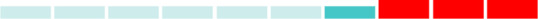

“Year” indicates the initial appearance of a burst keyword; “Strength” represents the degree of the burst; “Begin” signifies the onset of the burst; and “End” marks its termination. “Red” means burst keywords duration years; “Blue” means the onset of the keywords but not burst; “Light blue” means the keywords not yet present.

### 3.9 Trend topics and thematic evolution

**Figure 8:**
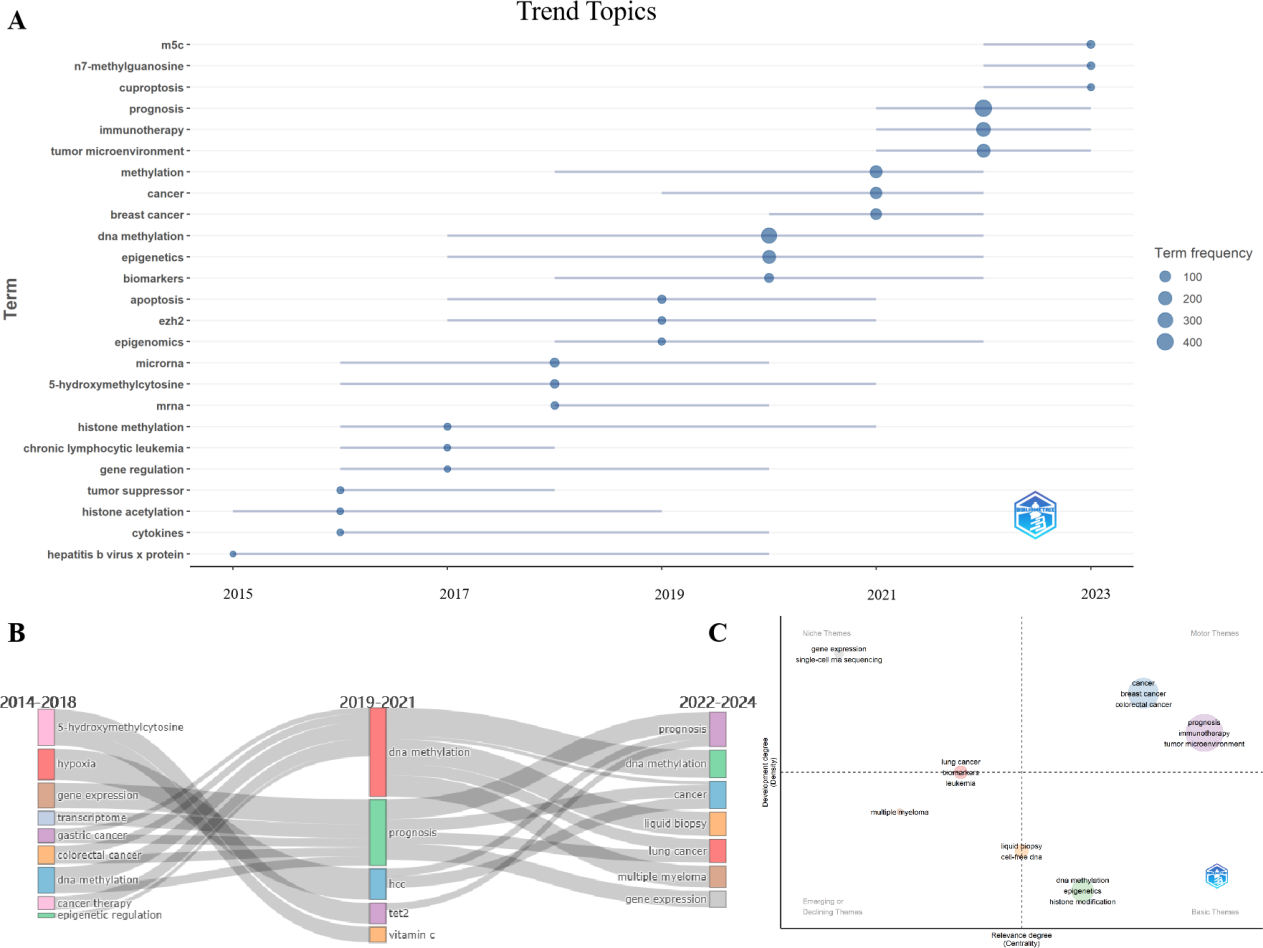
Trend topics and Thematic evolution. **(A)** represents changes in the top three hot topics across different eras. **(B, C)** represent changes in themes across the three phases, and research hotspots after 2022, where higher centrality and density suggest popular research topics with a greater number of articles.

We conducted a Trend Topics analysis on the included literature and identified the hot topics emerging after 2021 as follows: m5C, n7-methylguanosine, Cuproptosis, prognosis, immunotherapy, and tumor microenvironment (**Figure 8A**). If we divide the research over the past decade into three phases based on these hot topics and conduct a Thematic Evolution analysis (**Figure 8B**), we find that prognosis research has persisted throughout these 10 years and is closely linked to emerging approaches such as liquid biopsy. According to the Centrality and Density, prognosis, immunotherapy, and tumor microenvironment have emerged as popular and extensively studied topics since 2021 (**Figure 8C**), differing from earlier research where some hotspots such as tet2 and transcription RNA have gradually been replaced (**Supplementary Figures 9A, B**). Utilizing the CiteSpace dual-map visualization tool, we observe that both citing and cited literature predominantly belong to the fields of molecular biology and immunology (**Supplementary Figure 10**), suggesting that future developments could benefit from the incorporation of a wider range of disciplines.

## 4 Discussions

### 4.1 General information

Our findings reveal a consistent increase in publications linking RNA methylation to intra-tumor immune cells over the past decade, suggesting this field has emerged as a research hotspot, peaking in 2022. Chinese authors and research institutions have made outstanding contributions to the advancements in this area, ranking prominently in terms of both the number of publications and author influence. Additionally, the significant international and inter-institutional collaborations within this field underscore its widespread global interest. Journals such as Frontiers in Immunology and Nature Communications, which respectively publish the highest number and most influential articles in this category, have played a pivotal role in disseminating research outcomes. The enduring vitality of this field can be attributed to its robust research foundation, with seminal works by Professor Chiappinelli KB in Cell and Professor Han DL in Nature being the most widely cited. The evolution of research in this field is evident from changes in keywords, shifting from foundational studies to the integration with emerging trends like immunotherapy and liquid biopsy, thereby catalyzing new research foci like m5C, n7-methylguanosine, cuproptosis, prognosis, immunotherapy, and tumor microenvironment, which are the hottest trend topics in recent years. Below, we delve into the latest research progress in this domain.

### 4.2 Hot topics

More than 170 types of RNA modifications have been discovered in prokaryotes and eukaryotes (9). RNA methylation accounts for more than half of these modifications and plays a crucial role in post-transcriptional gene regulation (29). The most extensively studied RNA methylations include N1-methyladenosine (m1A), N6-methyladenosine (m6A), 5-methylcytidine (m5C), 7-methylguanosine (m7G), and 3-methylcytidine (m3C) (2). RNA methylation is a dynamic equilibrium process involving “writers” that catalyze the modification, “readers” that recognize the modification, and “erasers” that remove it (3), with an increasingly large family of proteins involved. These modifications regulate not only mRNA but also tRNA, lncRNA, sRNA, siRNA, snRNA, snoRNA, and have been reported to play roles in both solid and hematological tumors (30, 31). The m6A is the predominant form of RNA methylation in human mRNA, affecting RNA stability, transport, splicing, and translation, thereby influencing the expression of target RNAs (30). m5C modifications are widely distributed across various RNA types (32), enhancing mRNA stability and structure, ensuring translational accuracy and tRNA fragment integrity, influencing termination codon translation in rRNA, and regulating the nuclear export of mature mRNA (33). In contrast, m1A modifications targeting mRNA and mitochondrial tRNA have been less studied. Although some functional proteins are shared with m6A, dedicated erasers and readers for m1A are still under investigation. m1A primarily affects RNA stability by influencing base pairing (34). m7G is typically found in the 5’ cap and internal sites of mRNA, influencing miRNA structure (35) and mRNA nuclear export and translation (36). m3C is a specific modification of eukaryotic tRNA, crucial for maintaining tRNA structure and function, though its functional understanding remains limited.

TME refers to the surrounding environment of tumor cells, including blood vessels, immune cells, fibroblasts, cytokines, extracellular matrix, etc. RNA methylation plays a critical role in maintaining the immune microenvironment (37). RNA methylation affects immune cell function and may be associated with tumor immune evasion mechanisms. Targeting these RNA methylation regulatory proteins holds promise for enhancing the efficacy of immunotherapy (37–39). In innate immunity, METTL3 influences macrophage polarization, promoting the shift of bone marrow-derived macrophages from M1 to M2 polarization via the NF-κB and STAT3 pathways, thereby enhancing tumor infiltration and leading to reduced therapeutic efficacy of PD-1 monoclonal antibodies, accelerating tumor progression and distant metastasis (40). High expression of YBX1 is associated with M2 macrophage infiltration and T-cell exhaustion, suggesting it as a potential target for immunotherapy (40). ALKBH3 reduces m1A methylation, increases the stability of macrophage colony-stimulating factor (CSF-1) mRNA, and promotes the progression of breast and ovarian cancers (41). The m7G methyltransferase METTL1 also promotes macrophage polarization and positively correlates with M2 macrophages (42). YTHDF1 enhances MHC-II expression on dendritic cells and increases interleukin-12 secretion, thereby strengthening the adaptive immune response (43). The m6A-YTHDF1 axis restricts dendritic cell activation, while the absence of YTHDF1 enhances antigen-presenting ability (23). The absence of METTL3 inhibits MDSC accumulation and immunosuppressive capacity, leading to increased infiltration of CD4+ and CD8+ T cells (44). METTL1 upregulates chemokines CXCL5 and CXCL8, resulting in MDSC accumulation and immunosuppression in HCC and ICC (45). In acquired immunity, inhibiting METTL3 reduces m6A methylation levels, hinders effector T-cell differentiation, and inhibits CD4+ T-cell responses (45). ALKBH5 decreases m6A methylation levels, enhances the stability of CXCL2 and IFN-γ mRNA, and maintains the immune function of CD4+ T cells (46). m1A methylation at position 58 of tRNA is also involved in T-cell activation (47). Overexpression of METTL3 promotes m6A modification of NLRC5, increases the proportion of CD8+ T cells, and inhibits the progression of endometrial cancer (48). The absence of YTHDF2 in Tregs promotes Treg cell apoptosis and inhibits tumor progression via the YTHDF2-m6A-NF-κB pathway (49). YTHDF2 can recognize m6A modifications on ACER2 and promotes DLBCL progression (50).

Below, we will delve into how RNA methylation affects immune cells, using the trend topic m5C derived from literature analysis as an example. The m5C methyltransferase NSUN2 mediates the upregulation of interleukin-17a (IL-17A) induced by hyperhomocysteinemia by methylating IL-17A mRNA and enhancing its translation in T lymphocytes (51). During the latent phase of HIV-1 infection, NSUN1 binds to the 5’ long terminal repeat (LTR) of HIV-1 TAR RNA and produces m5C methylation. The binding of NSUN1 to TAR competes with TAR-TAR interactions, leading to the inhibition of HIV-1 transcription elongation and viral latency in CD4+ T cells (52). In patients with systemic lupus erythematosus (SLE), the levels of m5C and the expression of NSUN2 are reduced in CD4+ T cells. The hypermethylation of m5C in SLE is closely related to immune and inflammatory-related pathways, including the immune system, cytokine signaling, and interferon (IFN) signaling (53). In the systemic delivery of nanoparticle formulations, m5C also holds great promise for regulating CD8+ T cell immunity and inflammation (54).

RNA methylation plays a pivotal role in regulating tumor immunosuppressive factors and modulating tumor immune evasion. m6A methylation enhances PD-1/PD-L1 expression and reduces the cytotoxicity of CD8+ T cells through the METTL3-JNK signaling pathway, leading to tumor immune escape (55). Additionally, overexpression of METTL16 (56) and deficiency of ALKBH5 or FTO (57) can also suppress PD-L1 expression. Increasing evidence suggests that combining PD-L1 inhibitors with RNA modification modulators can enhance the efficacy of immunotherapy (58). For instance, YTHDF1 induces the transformation of immunologically “cold” tumors into “hot” tumors by promoting the degradation of MHC-I molecules (59). IGF2BP1 enhances the stability of PD-L1 mRNA, promoting tumor immune evasion, and can inhibit the proliferation and invasion of HCC cells (60). Several small molecule inhibitors with synergistic effects on immunotherapy have been developed. Examples include the METTL3 inhibitor STM2457 in AML (61) and cervical cancer (14), as well as CS1 and CS2 in AML (62) and the FTO inhibitor 18097 in breast cancer (63). The combination of RNA methylation inhibitors and immunotherapy drugs holds promise for significant breakthroughs and offers new treatment options for cancer patients.

Similarly, let’s revisit the impact of RNA methylation, with a particular emphasis on m5C, on tumor immune responses and the immune microenvironment. The m5C methyltransferase NSUN2 stabilizes TREX2 mRNA, reducing the infiltration of CD8+ T cells and increasing resistance to PD-L1 immunotherapy through the cGAS/STING pathway (64). m5C regulates prostate cancer genes and influences the roles of immature B cells, CD8+ T cells, M1 macrophages, and M2 macrophages in the tumor microenvironment (TME), as well as gene expression in different environments, providing clues for prognosis prediction (65). Speaking of prognosis, studies have established prognostic models in cervical cancer using m6A/m5C/m1A-related genes to predict survival time and its correlation with immune cell infiltration, and found associations with sensitivity to anti-CTLA-4 immunotherapy drugs (66). Meanwhile, based on the degree of tumor immune cell infiltration and different m5C regulators, it has been discovered that NSUN3 is closely related to CD8+ T cells, while NSUN4 is closely related to neutrophils, confirming that m5C can regulate the tumor immune microenvironment to predict the prognosis of lung squamous cell carcinoma (67). In hematological tumors, it has also been found that m5C genes are not only associated with immune cell infiltration characteristics but also with patient prognosis. The developed m5C score can serve as a reliable indicator for AML prognosis, and the prognostic value of the m5C score has been validated from the perspectives of drug resistance and immunotherapy (68).

The regulation of RNA methylation ultimately leads to recognition by RNA-binding proteins (RBPs), which play a crucial role in directly affecting tumor immune cells or downstream genes. YTHDF1 mediates m6A-dependent translational regulation, thereby stimulating T-cell activation and enhancing cytokine production induced by TLR4/NF-κB signaling (69). YTHDF1 recognizes m6A-modified RNA encoding lysosomal proteases, increasing the translation of lysosomal proteases in dendritic cells, which in turn inhibits CD8+ T cells and enhances the therapeutic effect of anti-PD-1 antibodies (70). YTHDF1 recognizes m6A modification of JAK1 mRNA in Tumor-infiltrating myeloid cells mediated by METTL3, and METTL3-m6A-YTHDF1 enhances the translation of JAK1 mRNA, subsequently promoting STAT3 phosphorylation and tumor growth in mice (71). YTHDF3 recognizes modification sites around the stop codon of FOXO3 mRNA by recruiting eIF3A and eIF4B, promoting FOXO3 translation and subsequently initiating autophagy (72). IGF2BP2 recruits proteins such as eIF4E and eIF3A to MYC, GPT2, and SLC1A5 mRNA, regulating glutamine metabolism in acute myeloid leukemia and modulating the immune response of macrophages through epigenetic reprogramming (73, 74).

Based on the above analysis, it can be observed that RNA methylation is associated with tumor prognosis by influencing immune cell infiltration. There are numerous reports on the role of RNA modifications in regulating tumor immune activity. For example, in acute myeloid leukemia (AML), inhibiting the demethylase FTO can suppress the expression of immune checkpoint molecules PD-L1 and LILRB4, inducing T-cell cytotoxicity against tumor cells (75). In melanoma cells, downregulation of FTO reduces the removal of m6A modifications on PD-1, affecting mRNA decay and inhibiting tumor growth (76). In tumor cells, METTL3 and METTL14 can inhibit the expression of CXCL9 and CXCL10. Their knockdown recruits CD8+ T cells and natural killer (NK) cells into the tumor microenvironment, enhancing the responsiveness to immunotherapy (77). IL-8, as a chemokine, participates in the recruitment and immunosuppression of tumor-associated macrophages (TAMs). ALKBH5 promotes the expression of the chemokine CXCL8/IL8 in human glioblastoma. Knockout of ALKBH5 leads to reduced TAMs, increased CD8+ T-cell infiltration, and increased tumor sensitivity to immunotherapy (78).

### 4.3 Limitations

This study leverages Bibliometric, VOSviewer, CiteSpace software to conduct a visual analysis of the literature, facilitating researchers in gaining an intuitive understanding of the developmental trends and academic frontiers within this field. Nonetheless, several limitations persist. Firstly, the inclusion of solely English articles and reviews from WOS introduces potential biases. Secondly, due to the limitations of the software’s functionality, some details may be overlooked during the analysis process, resulting in insufficient textual representation in the generated visualizations. Lastly, to facilitate statistical convenience, outstanding articles published in 2024 may be inadvertently excluded. Taking into account the differences in database recognition across different regions of the world, for subsequent analyses, we can further incorporate relevant studies from other databases such as PubMed and Scopus, and include as many recent articles as possible to make the presentation of research trends more cutting-edge.

## 5 Conclusion

This study employs bibliometric analysis to statistically quantify and visually analyze literature related to RNA methylation and tumor immune cells. It reveals insights into the research landscape, including contributions from authors, institutions, and countries, as well as the popular journals that publish in this field. Furthermore, through the analysis of keywords and the evolution of research, the study identifies the hotspots and current status of research within this domain.

## Data Availability

The original contributions presented in the study are included in the article/**Supplementary Material**. Further inquiries can be directed to the corresponding author.
