# Supplementary material for: Bibliometric analysis and visualization of the research on the relationship between RNA methylation and immune cell infiltration in tumors
Source: Front Immunol. 2024 Dec 12;15:1477828. doi: 10.3389/fimmu.2024.1477828 (PMC11669668; doi:10.3389/fimmu.2024.1477828)
Supplement: Supplementary file 2 [file Table1.docx]

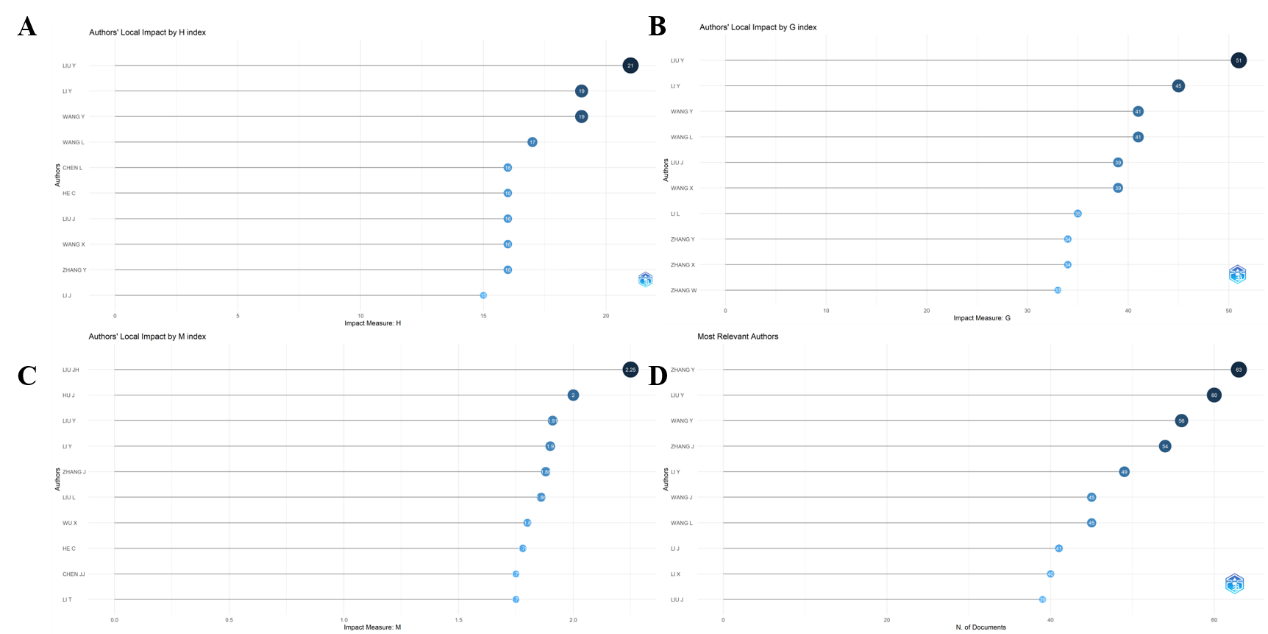


Supplementary Figure 1 Top 10 author about h-index, g-index, m-index and publication.


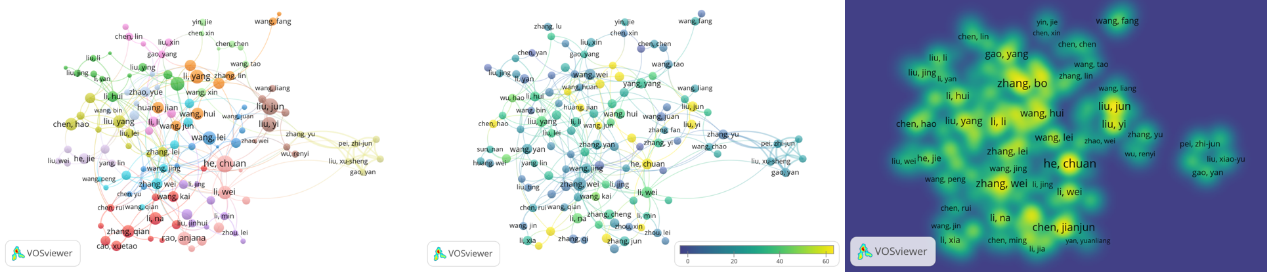


Supplementary Figure 2 Co-authorship analysis by citation.


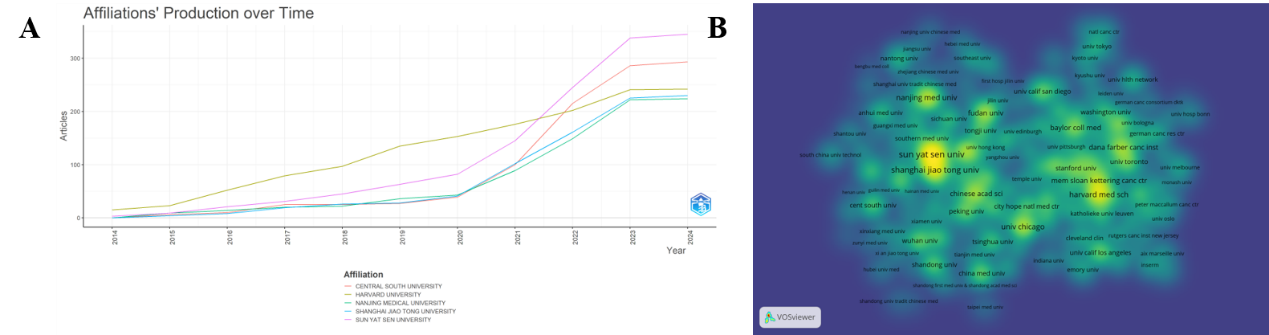


Supplementary Figure 3 Co-organization analysis by citation.


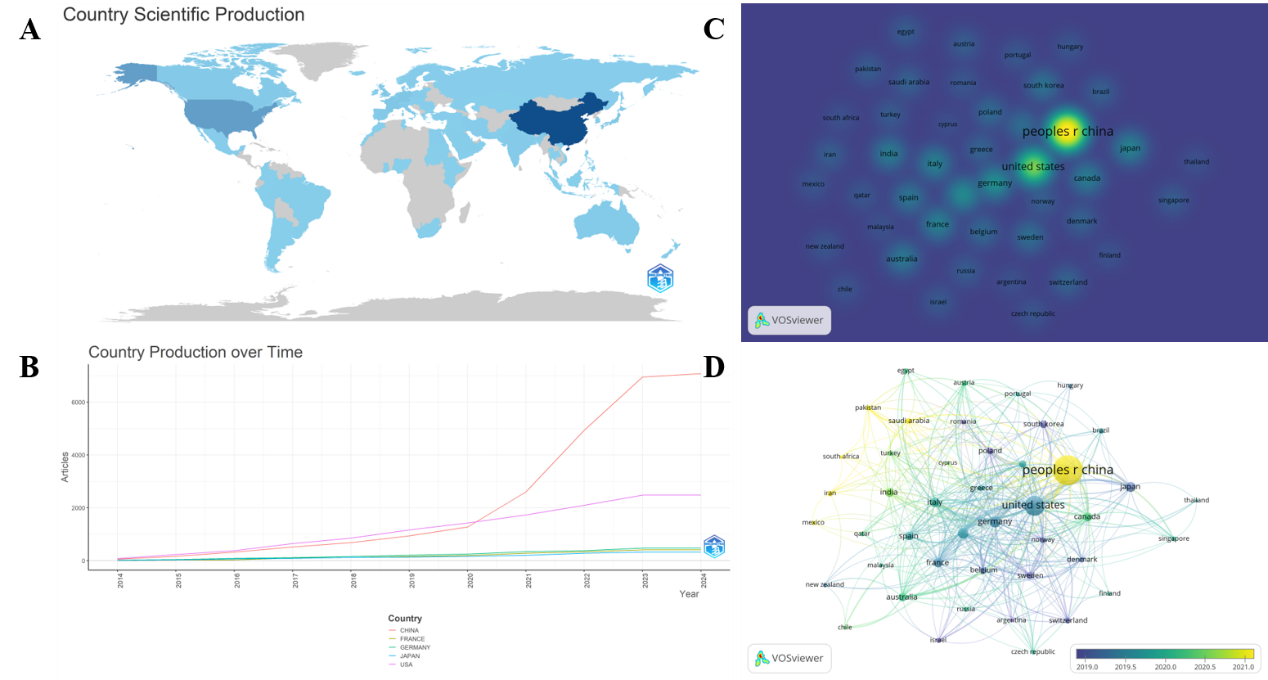


Supplementary Figure 4 Country collaboration analysis by citation.


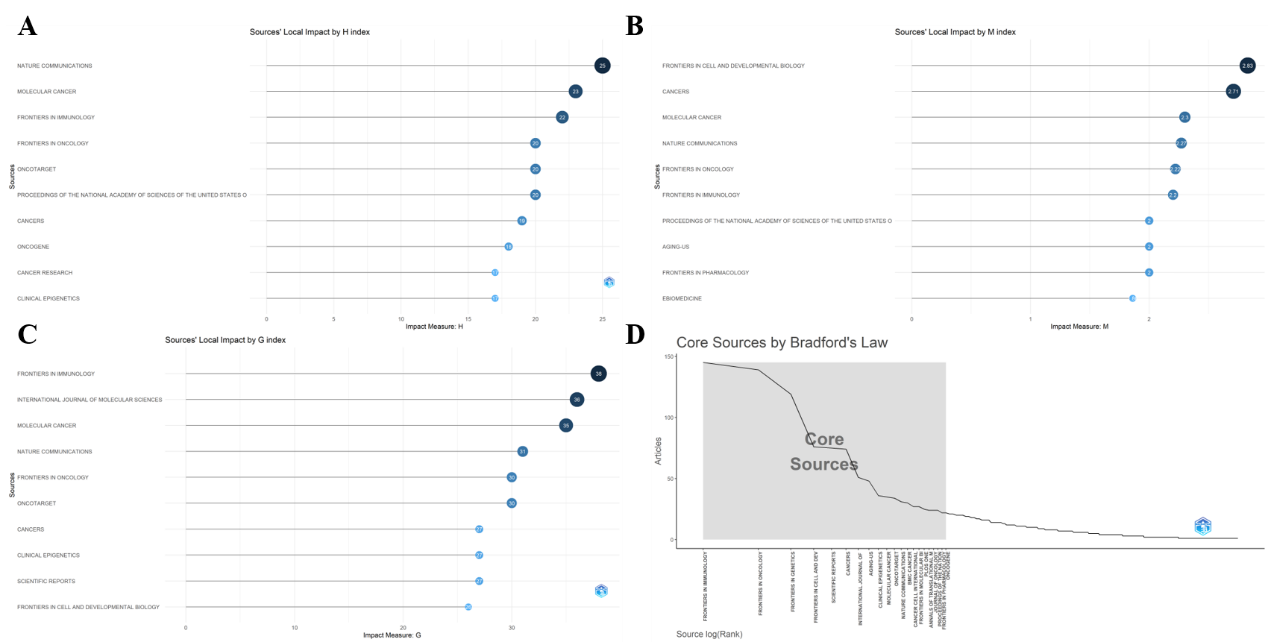


Supplementary Figure 5 Journal impact analysis.


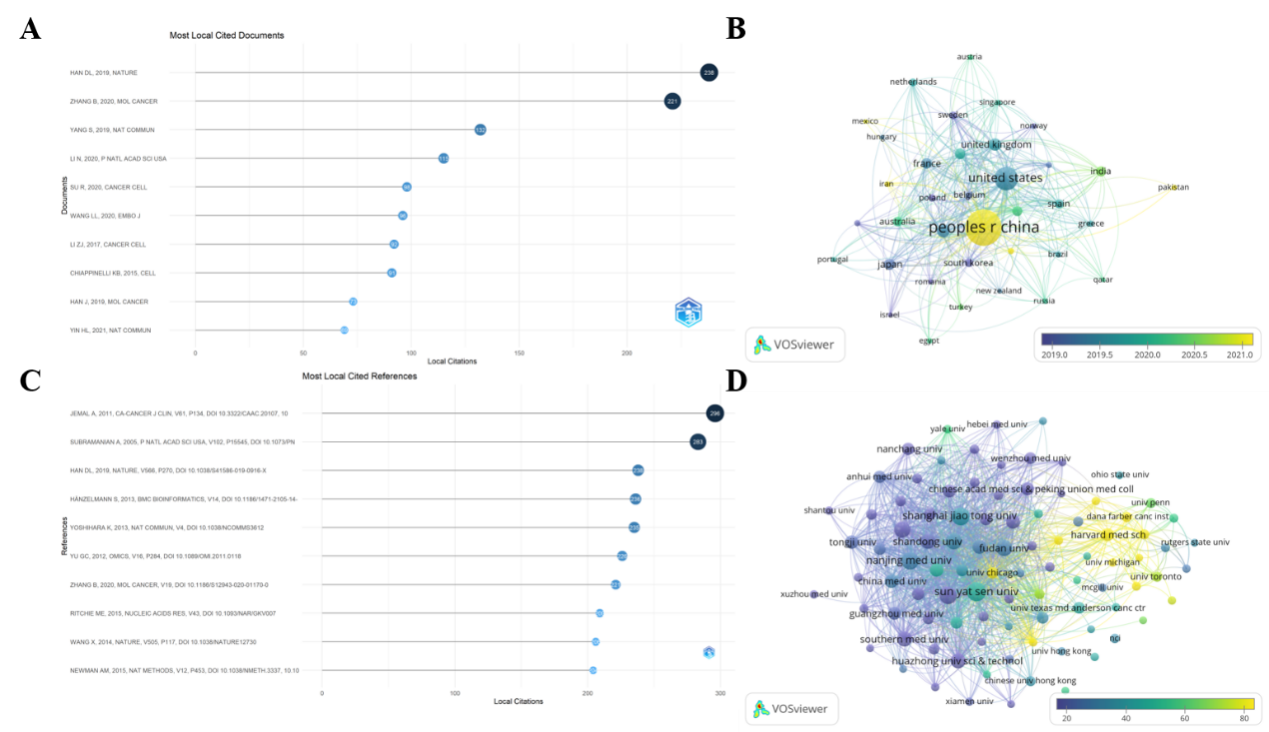


Supplementary Figure 6 Analysis of authors, countries, institutions, and references of highly cited articles.


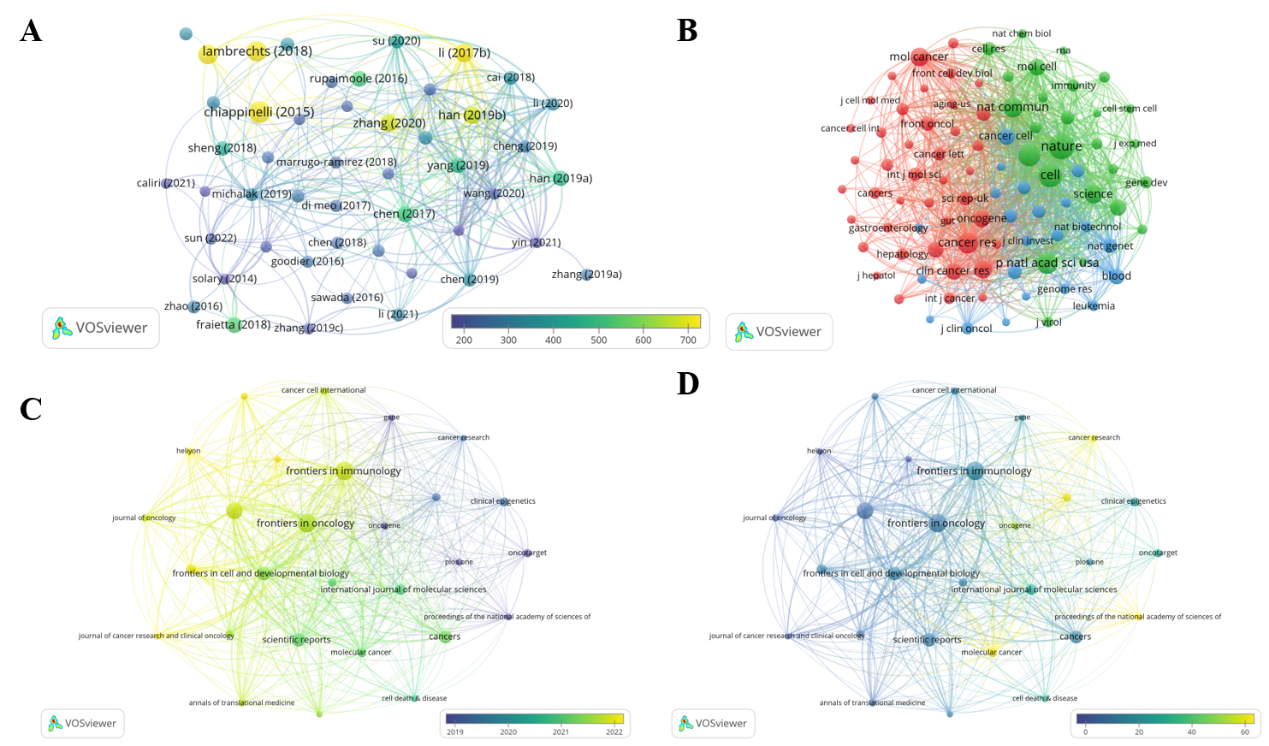


Supplementary Figure 7 Co-citation and document coupling analysis.


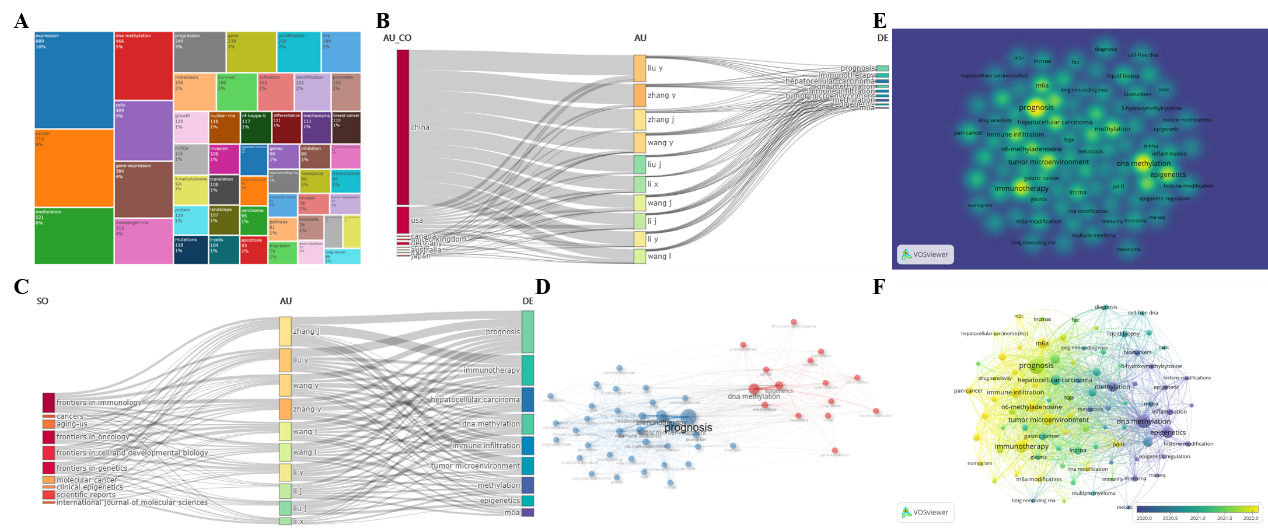


Supplementary Figure 8 Popular keywords and the connections between them.


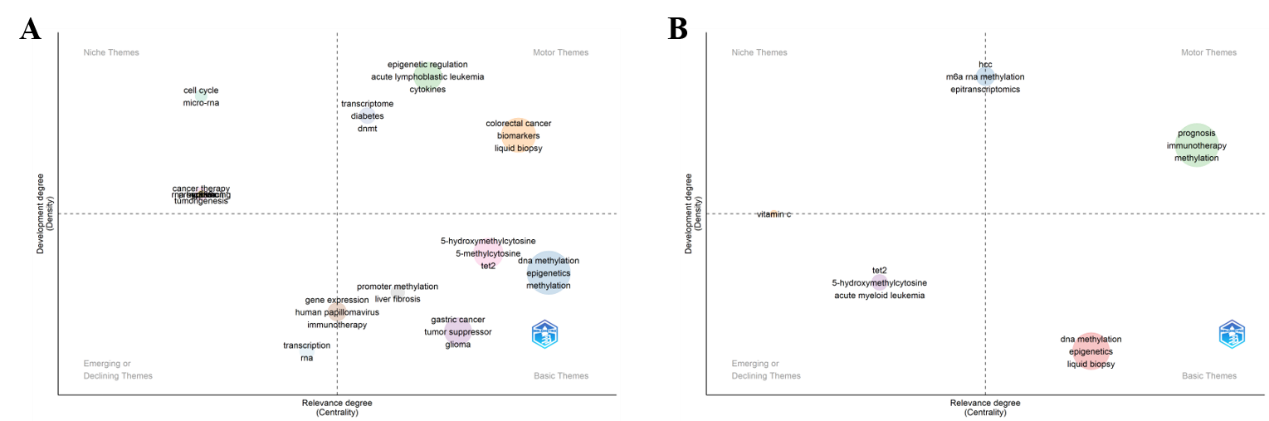


Supplementary Figure 9 Thematic Evolution-Map 2014-2018, 2019-2021.


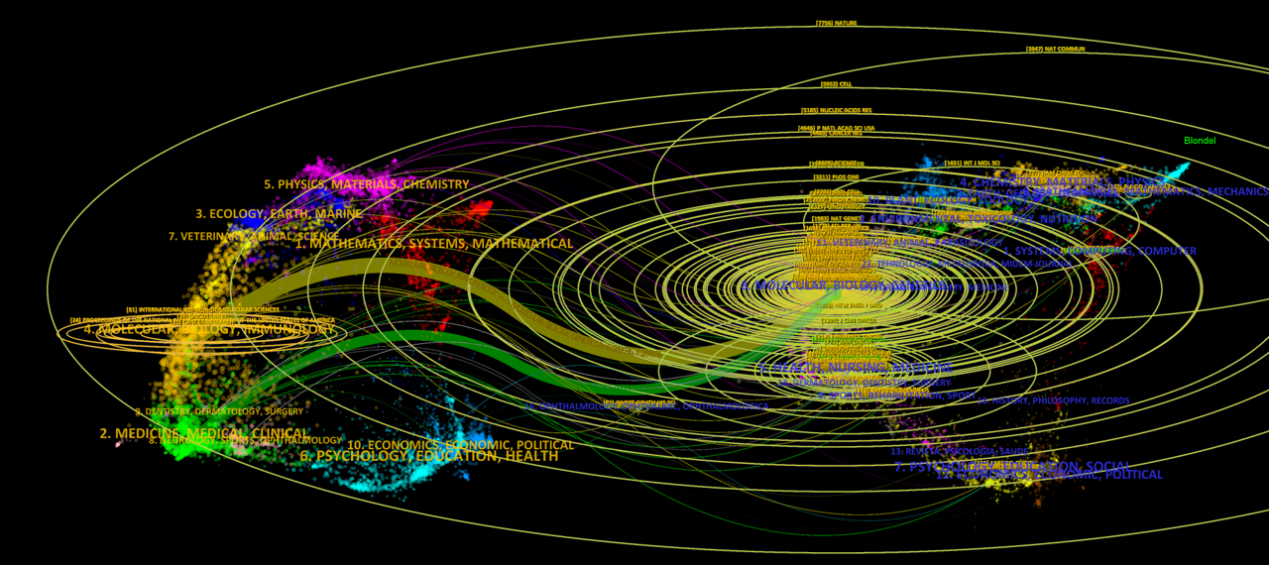


Supplementary Figure 10 Dual-Map.
